# Orally Co-Infected *Aedes albopictus* from La Reunion Island, Indian Ocean, Can Deliver Both Dengue and Chikungunya Infectious Viral Particles in Their Saliva

**DOI:** 10.1371/journal.pntd.0000706

**Published:** 2010-06-08

**Authors:** Marie Vazeille, Laurence Mousson, Estelle Martin, Anna-Bella Failloux

**Affiliations:** Institut Pasteur, Génétique moléculaire des Bunyavirus, Paris, France; Centers for Disease Control and Prevention, United States of America

## Abstract

**Background:**

First described in humans in 1964, reports of co-infections with dengue (DENV) and chikungunya (CHIKV) viruses are increasing, particularly after the emergence of chikungunya (CHIK) in the Indian Ocean in 2005–2006 due to a new variant highly transmitted by *Aedes albopictus*. In this geographic area, a dengue (DEN) outbreak transmitted by *Ae. albopictus* took place shortly before the emergence of CHIK and co-infections were reported in patients. A co-infection in humans can occur following the bite of two mosquitoes infected with one virus or to the bite of a mosquito infected with two viruses. Co-infections in mosquitoes have never been demonstrated in the field or in the laboratory. Thus, we question about the ability of a mosquito to deliver infectious particles of two different viruses through the female saliva.

**Methodology/Principal Findings:**

We orally exposed *Ae. albopictus* from La Reunion Island with DENV-1 and CHIKV isolated respectively during the 2004–2005 and the 2005–2006 outbreaks on this same island. We were able to show that *Ae. albopictus* could disseminate both viruses and deliver both infectious viral particles concomitantly in its saliva. We also succeeded in inducing a secondary infection with CHIKV in mosquitoes previously inoculated with DENV-1.

**Conclusions/Significance:**

In this study, we underline the ability of *Ae. albopictus* to be orally co-infected with two different arboviruses and furthermore, its capacity to deliver concomitantly infectious particles of CHIKV and DENV in saliva. This finding is of particular concern as *Ae. albopictus* is still expanding its geographical range in the tropical as well as in the temperate regions. Further studies are needed to try to elucidate the molecular/cellular basis of this phenomenon.

## Author Summary

Dengue (DEN) and chikungunya (CHIK) are two mosquito borne infections transmitted by *Aedes* mosquitoes in the tropical world. *Ae. albopictus* has been shown to efficiently transmit the new variant of CHIK virus (CHIKV) that emerged in the Indian Ocean region in 2005. At the same time, this vector is able to sustain outbreaks due to DEN virus (DENV). Since this CHIK emergence, co-infections DENV-CHIKV in humans have been regularly reported. This phenomenon, known for a long time, may be due to two consecutive bites from two mosquitoes infected by one virus or by the bite of a mosquito infected by both viruses. We used two viral strains isolated in La Reunion Island, DENV-1 in 2004 and CHIKV in 2005, and co-infected an autochthonous strain of *Ae. albopictus*, testing experimentally one of the possible ways to get co-infections in humans. We were able to show the ability of *Ae. albopictus* to replicate simultaneously both arboviruses and, furthermore, to deliver both infectious viral particles concomitantly in their saliva. This finding is of particular interest since *Ae. albopictus* is now widely distributed all around the world and still expanding its geographical range.

## Introduction

Dengue (DEN) and chikungunya (CHIK) are two mosquito-borne viral infections transmitted by mosquitoes of the genus *Aedes*. Dengue viruses (DENV-1, -2, -3, -4) belonging to the *Flaviviridae* family, *Flavivirus* genus [1] are of primarily concern as they are responsible of the most important arboviral disease widely distributed in the tropical world [2]. Dengue infection may be unapparent or induce an undifferentiated febrile illness, a classic dengue fever (DF), or a dengue hemorrhagic fever (DHF). The highest prevalence of DEN is observed in South-East Asia and South Americas with approximately 50–100 million cases and 25 000 deaths per year [3]. The transmission is mainly ensured by the highly anthropophilic *Aedes aegypti* in urban areas [4]. However, *Aedes albopictus* may act as a secondary vector in rural areas and even as the main vector when *Ae. aegypti* is not present or too scarce as observed in some localities in China, Japan, Hawaii, and Seychelles [5]. *Ae. albopictus* was indeed the only vector in the recent dengue outbreaks observed in the Indian Ocean on La Reunion Island [6] and Madagascar [7].

Chikungunya virus (CHIKV), first isolated in Tanzania in 1953 [8], belongs to the *Togaviridae* family, *Alphavirus* genus [9] and is endemic to Africa, India and South-East Asia. In Africa, the virus is maintained within a sylvatic cycle with wild mosquitoes (*Aedes furcifer*, *Aedes luteocephalus*, *Aedes taylori*, *Aedes africanus*) feeding preferentially on primates [10], [11]. In Asia, CHIKV is mainly transmitted within an urban cycle in an inter-human transmission achieved essentially by the human-biting *Ae. aegypti* and the less anthropophilic *Ae. albopictus*, which prefers suburban and rural areas where it colonizes both artificial and natural containers [12], [13]. CHIKV mainly induces high fever and severe arthralgia, and had limited impacts on public health before its emergence in the Indian Ocean in 2005. This major epidemic started in the Comoro Islands in January 2005 then spread rapidly to the other islands of the region, Mayotte, Seychelles, La Reunion and Mauritius [14]. In April 2006, one third of the population in La Reunion Island had been in contact with the virus [15]. Surprisingly, the vector in this epidemic was not *Ae. aegypti*, only present as residual populations on the island, but *Ae. albopictus* [16]–[18]. This latter species was proved to be a very efficient vector of a mutated strain CHIKV harboring a switch from an alanine to a valine in the E1 glycoprotein, mutation that appeared in the course of the outbreak and was then selected as a major epidemic genotype [19], [14]. CHIK outbreaks spread rapidly and caused several million clinical cases in the Indian Ocean Islands and India, where outbreaks had been absent for 32 years [20], [21]. One consequence was an increasing overlapping in the distribution of the two arboviral diseases, DEN and CHIK leading to the detection of a higher number of co-infections in humans.

Co-infection by DENV and CHIKV in patients has been known for a long time. First described in 1964 in South India [22], it has been more frequently reported since the re-emergence of CHIK: Sri-Lanka [23], India [24], [25], Malaysia [26] where the main vector involved is *Ae. aegypti* and in Gabon [27], Madagascar [7] and La Reunion Island [28] where viral transmission is achieved by *Ae. albopictus.* On La Reunion Island, the CHIK outbreak was preceded by a small outbreak due to DENV-1 with 228 cases reported between March and July 2004 [6]. Despite the limited impact of the DEN outbreak, in January 2006, suspected cases of co-infection DENV-CHIKV in patients were reported [28] and the same phenomenon was also observed in Madagascar in January 2006 [7].

Dual arboviral infections in humans can occur following the bite of two mosquitoes, each infected by one virus, or the bite of one mosquito infected by the two viruses. If isolations of either virus from mosquitoes collected in the course of an epidemic have been already noticed, to our knowledge, doubly infected mosquitoes have never been described. A recent study even concluded on the failure to prove co-infection of *Ae. aegypti* by these two viruses [29]. Here, we orally infected in a single blood-meal, *Ae. albopictus* from La Reunion Island with autochthonous viral strains isolated during the 2004–2005 outbreak of DEN-1 and the 2005–2006 CHIK outbreak. Dissemination of both viruses within the vector was checked by immunofluorescence assay on female head squashes. Furthermore, saliva was collected from each female to check their ability to deliver simultaneously both DENV and CHIKV infectious particles. Additionally, we performed secondary infections by inoculating first DENV-1 then providing CHIKV in an infectious blood-meal.

## Methods

### Ethics statement

All experiments on live vertebrates were performed in compliance with French and European regulation and according to the Institut Pasteur guidelines for laboratory animal husbandry and care.

### Mosquitoes

*Ae. albopictus* Providence (ALPROV) were collected as eggs in 2006 on La Reunion Island and provided by the DRASS (Direction Régionale des Affaires Sanitaires et Sociales). The F5 or F6 generation was used for experimental infections. Colonies were maintained at 28±1°C with a light:dark cycle of 16 h:8 h and a 80% relative humidity. Larvae were reared in pans containing 1 yeast tablet in 1 liter of tap water. Adults were provided with 10% sucrose solution *ad libitum* and fed three times a week on anaesthetized mice.

### Virus

The CHIKV 06.21 isolated in November 2005 from a new-born male from La Reunion Island presenting meningo-encephalitis symptoms was used for all experiments. This strain harbored the A->V mutation at the position 226 in the E1 glycoprotein (E1-226V) [14]. Viral stock used was a third passage on *Ae. albopictus* C6/36 [30] stored at −80°C in aliquots. Procedure for C6/36 cell infections and passages are described elsewhere [19].

The DENV-1 185/04 was isolated in May 2004 from the plasma of a patient in La Reunion Island. The strain belonged to the Brazil group of the Pacific genotype which was the main genotype isolated during the outbreak (GenBank: DQ285558.1). The virus was provided as a second passage on C6/36 cells. DENV-1 production on mosquito cell cultures being insufficient to allow mosquito oral infections, the virus stock was produced by inoculating intra-thoracically mosquitoes with the viral strain [31]. Inoculated mosquitoes were incubated 10 days at 28°C and their infectious status checked by indirect immunofluorescent assay (IFA) on head squashes [32]. Bodies were then pooled and triturated in heated (56°C for 30 min) FCS (Fetal Calf Serum). The supernatant fluid recovered after low speed centrifugation was used as a source of virus in mosquito blood-meals.

Both viruses were provided by the French National Reference Center for Arboviruses at the Institut Pasteur which had obtained the verbal consent from patients or parent's patients who provided blood sera.

### Intrathoracic inoculation of mosquitoes

Adult females were inoculated using the protocol described by Rosen and Gubler [31], each mosquito receiving 0.17 µL (i.e. 10^2.8^ PFU/mL) of the DENV-1 strain.

### Oral infection of mosquitoes

Infection assays were performed with 7 day-old females which were allowed to feed for 15 min through a chicken skin membrane covering the base of a glass feeder containing the blood-virus mixture maintained at 37°C. The infectious blood-meal was composed of a virus suspension diluted (1∶3) in washed rabbit erythrocytes isolated from arterial blood collected 24 h before the infectious blood-meal [33]. A phagostimulant, ATP, was added at a final concentration of 5×10^−3^ M. Fully engorged females were transferred to small cardboard containers and maintained with 10% sucrose at 28±1°C for 14 days. Viral suspension provided in the blood-meal contained one or two viruses. For the secondary infection experiment, the blood-meal with CHIKV yielding 10^6^ FFU (foci forming unit)/mL was provided 7 or 13 days after inoculation with DENV-1. As control, females were inoculated with DENV-1 alone or orally infected with CHIKV alone. For the two trials where both viruses were provided by oral route, titers of the blood-meals were respectively: 10^6^ FFU/mL for CHIKV in both trials and 10^4,5^ FFU/mL for DENV-1 in the trial 1 and 10^5,9^ FFU/mL for DENV-1 in the trial 2.

### Saliva collection and virus detection/titration

At day 14 post-infection, females were chilled, and their wings and legs removed, the stress inducing a forced salivation. Proboscis was inserted into 1 µL micropipette (microcaps®, Drummond Scientific Company, USA) filled with FCS. After 45 min, medium containing the saliva was expelled under pressure into 1.5 mL tubes containing Leibovitz L15 medium supplemented with 10% FBS. To allow a specific detection of both viruses, each sample was inoculated in two wells, one for the detection of CHIKV and one for the detection of DENV-1. 20 µL of each sample were added to monolayers of C6/36 cells in 24 wells plaque to detect infectious particles by the foci forming technique using an immunoperoxydase assay. Cells were incubated 3 days for CHIKV and 5 days for DENV-1 at 28°C under an overlay consisting of 50% of Leibovitz L-15 medium supplemented with 10% FBS and 50% of carboxyl methyl cellulose. Cells were then fixed with 3.6% formaldehyde at room temperature (RT) for 20 min and a immunoperoxydase assay staining was performed to detect foci. After a first incubation of 4 min with PBS 0.1% Triton X-100 (Sigma) at RT, cells were incubated 20 min at 37°C with a mouse ascitic fluid at a dilution of 1∶1000 for CHIKV and 1∶100 for DENV-1 (both ascetic fluids were provided by the French National Reference Center for Arbovirus at the Institut Pasteur). After a wash in PBS 1X, cells were incubated at 37°C for 45 min with peroxydase-conjugated goat anti-mouse IgG antibody (Pierce biotechnology, Rockford, USA) at a 1∶100 dilution in PBS 1X. After final wash in PBS 1X, Fast 3,3′ Diaminobenzidine (Sigma) was used to reveal the staining and foci were counted. The titer of infectious particles per saliva was expressed as FFU/mL (mean ± standard deviation).

### Female status analyzed by IFA

After salivation, females were tested for the presence of CHIKV and DENV-1 by IFA on their head squashes [32]. CHIKV and DENV-1 antigens were detected with the same mouse ascitic fluids used for saliva titration. Head squashes being performed between two slides, infection status of females fed with both viruses, could be checked for both antigens by using one slide for each IFA. Mosquitoes inoculated with CHIKV and DENV-1 were used as positive controls, negative controls were inoculated with cell culture media.

## Results

### Secondary infection with CHIKV of *Ae. albopictus* previously inoculated with DENV-1

As shown on Table 1, only few females inoculated with DENV-1 were willing to feed on an artificial blood-meal in the BSL-3 insectarium. When a blood-meal was proposed at day 7 post-inoculation, eight females out of 106 fed and among them, three survived until day 13 post-inoculation. By IFA on head squashes, we found that all 3 females had disseminated both viruses. Besides, when a blood-meal was offered at day 13 post-inoculation, eight females out of 54 females fed and four survived until day 20 post-inoculation. These four females had disseminated both viruses. Control females inoculated or orally infected by only one virus were all positive.

**Table 1:** Superinfection with CHIKV of *Aedes albopictus* Providence previously inoculated with DENV-1.

at day 7 post-inoculation			at day 13 post-inoculation		
Engorged females at day 7 pi (N)	Surviving females at day 13 pi (N)	Co-infected females at day 13 pi (N)	Engorged females at day 13 pi (N)	Surviving females at day 20 pi (N)	Co-infected females at day 20 pi (N)
8 (106)	3 (8)	3 (3)	8 (54)	4 (8)	4 (4)

N, number of females tested.

### Oral co-infections

Females were exposed to both viruses in a same blood-meal and disseminated infection rates were estimated at day 14 post-infection (pi) (Table 2). In the trial 1, 71.6% of females have only disseminated CHIKV, 0% only DENV-1, 18.6% both viruses and 9.8% did not disseminate any virus. In the trial 2, 30.8% of females have only disseminated CHIKV, 7.7% only DENV-1, 50.8% both viruses and 10.7% did not disseminate any virus. When providing a higher titer of DENV-1 in the blood-meal (trial 2), a higher proportion of females were co-infected with both viruses.

**Table 2:** Disseminated infection rates (%) of *Aedes albopictus* Providence 14 days after a blood-meal with both CHIKV and DENV-1.

	CHIKV	DENV-1	CHIKV + DENV-1	Non-infected	Total
Trial 1	71.6	0	18.6	9.8	102
Trial 2	30.8	7.7	50.8	10.7	65

Disseminated infection rates were estimated by IFA on head squashes. For CHIKV, both trials used a blood-meal at a titer of 10^6^ FFU/mL. For DENV-1, trial 1 corresponded to a blood-meal at a titer of 10^4.5^ FFU/mL and trial 2 to a titer of 10^5.9^ FFU/mL. Total corresponds to the total number of females tested.

For females having disseminated both viruses, saliva was collected at day 14 pi and titrated. Relative transmission of the two viruses are shown in Table 3. In the trial 1, out of 19 saliva, 4 contained simultaneously CHIKV and DENV-1, 4 only CHIKV, 3 only DENV-1 and 8 no virus. In the trial 2, out of 33 saliva, 9 presented concomitantly CHIKV and DENV-1, 8 only CHIKV, 2 only DENV-1 and 14 no virus. Mean titers, expressed as FFU per saliva, and standard deviation are shown on Table 4.

**Table 3:** Relative transmission of the two viruses by dually infected mosquitoes.

		DENV-1			
		Trial 1		Trial 2	
		Yes	No	Yes	No
CHIKV	Yes	4	4	9	8
	No	3	8	2	14

Saliva from females having disseminated both viruses 14 days after an infectious blood-meal was tested for the presence of both viruses. For CHIKV, both trials used a blood-meal at a titer of 10^6^ FFU/mL. For DENV-1, trial 1 corresponded to a blood-meal at a titer of 10^4.5^ FFU/mL and trial 2 to a titer of 10^5.9^ FFU/mL.

**Table 4:** Mean numbers ± standard deviations of infectious viral particles in saliva of *Aedes albopictus* Providence co-infected with CHIKV and DENV-1.

		Trial 1 (N)	Trial 2 (N)
Saliva with both viruses detected	CHIKV	16±10 (4)	44±75 (9)
	DENV-1	74±134 (4)	46±66 (9)
Saliva with only one virus detected	CHIKV	4±1 (4)	45±64 (8)
	DENV-1	4±1 (3)	17±0 (2)

The saliva of females detected positive for both viruses by IFA on head squashes, 14 days after an infectious blood-meal were titrated. Titers were expressed in FFU (foci forming units). For CHIKV, both trials used a blood-meal at a titer of 10^6^ FFU/mL. For DENV-1, trial 1 corresponded to a blood-meal at a titer of 10^4.5^ FFU/mL and trial 2 to a titer of 10^5.9^ FFU/mL. N refers to the total number of females tested.

## Discussion

We report here the ability of *Ae. albopictus* from La Reunion Island to replicate simultaneously autochthonous strains of DENV-1 and CHIKV provided in the same blood-meal and to deliver both infectious viral particles in their saliva. To our knowledge, such co-infection has never been shown neither under laboratory conditions nor in the field. Lastly, we succeeded in inducing a secondary infection with CHIKV 7 or 13 days after a first infection with DENV-1 virus.

CHIKV and DENV are both transmitted by *Ae. aegypti* and *Ae. albopictus*, the former being considered the major vector and the latter, the secondary vector. However *Ae. albopictus* is able to sustain DEN outbreaks in the absence of *Ae. aegypti* [5]. Indeed, in the Indian Ocean, *Ae. albopictus* was predominant in Seychelles and in La Reunion Island where the species took part of DEN outbreaks in 1976-77 [34], [35] and in 2005 [6]. On La Reunion Island, *Ae. aegypti* populations are scarce and do not exhibit a high anthropophily [16], [17] while *Ae. albopictus* has favored the emergence of a new CHIKV strain harboring a substitution (alanine → valine) at the position 226 of the E1 glycoprotein during the 2005–2006 outbreak. This variant presents high levels of replication in *Ae. albopictus* [19] and a short extrinsic incubation period as the virus could be found in saliva as early as two days after infection [36]. Subsequent outbreaks due to the new CHIKV variant were often related to transmission by *Ae. albopictus* corroborating an adaptative mutation in response to a requirement for transmission by this species. Moreover, co-infections with both DENV and CHIKV have been detected in some patients from La Reunion Island [28] and Madagascar in 2006 [7].

Co-infections CHIKV-DENV in patients have been first described in 1967 and since the emergence of CHIKV in the Indian Ocean, reports of co-infections are increasing: in the Indian Ocean, as mentioned above, but also in Sri-Lanka [23], Malaysia [26], in India [24], [25] where the main vector involved is *Ae. aegypti* and in Gabon [27]. Except for the Americas still free of CHIK infection, the geographic range of CHIK is now largely overlapping that of DEN. Furthermore, the emergence of CHIK outbreaks due to the new variant coincided with the recent establishment of *Ae. albopictus* in Central Africa, in Cameroon [37], [38] and Gabon [39], [40]. In 2007, patients with co-infections DENV-CHIKV were indeed detected for the first time in Africa in Gabon [27].

Co-infection of a mosquito vector by two different viruses can occur by the way of two successive infectious blood-meals taken on two different viremic hosts or by one blood-meal taken on a co-infected host. We chose to mix both viruses in the same meal since *Ae. albopictus* females from La Reunion Island were reluctant to feed twice at 7 or 13 days interval on an infectious meal in the BSL-3 conditions. If *Ae. albopictus* females can be readily fed on an artificial meal in a BSL-3 laboratory it is very difficult to make them take a second artificial meal in the same conditions after 1 or 2 weeks of incubation and even to make then take a first meal if they have been kept more than 48 h in the depression conditions of the BSL-3. Temperature and humidity were optimal, females had the opportunity to lay their eggs if needed, were starved prior blood-feeding as usual. Therefore, we were unable to offer viruses in sequential meals and choose to test the possibility of a superinfection by first injecting one virus (DENV-1) then offering an infectious blood meal with the second virus (CHIKV) 7 or 13 days latter. Even then, we had very few individuals blood-fed but could however detect replication and dissemination of both viruses.

*Ae. albopictus* from La Reunion Island were already known to be a very efficient vector for the new CHIKV variant and able to sustain DEN outbreaks. We demonstrated that replication of both viruses can take place simultaneously and vectors become able to transmit the two viruses in a single bite. In *Ae. albopictus*, CHIKV infectious particles can be found in saliva from two days after oral infection [36] when this delay is much longer for DENV at least 10 days [41]–[42]. We performed the saliva analysis 14 days after feeding the females on a co-infected blood-meal but were not able to detect infectious viral particles in all saliva collected from females presenting a disseminated infection of both viruses. As CHIKV was not detected, when it should have been considering its rapid excretion in saliva [36], females with negative saliva must have been females unable to salivate using our technique [36] or excreting a very small amount of infectious particles that we could not detect due to the low sensitivity of the technique of viral detection. It would be interesting to perform a kinetic study of excretion of infectious particles from females exposed to one or both viruses while controlling the presence of saliva in the collected samples. We performed our study with *Ae. albopictus* from La Reunion Island with two viral strains isolated in the same area during the DENV-1 outbreak of 2005 and the CHIKV outbreak of 2005–2006. This combination vector/virus fits well with the natural context strengthening the findings of this work. Nevertheless, it seems difficult to infer to other epidemiological contexts as shown by the failure of Rohani *et al.* [29] to prove co-infection of *Ae. aegypti* by CHIKV and DENV. The mechanism of co-infection in *Ae. albopictus* by two different arboviruses needs to be further investigated and tested using additional trials with different viral strains to determine if this phenomenon is an exception due to the particularly well adapted partners or a quite common mechanism. The still ongoing expansion of this species, particularly in Africa where numerous arboviruses are transmitted, in of particular concern and, if the co-infection was a quite usual phenomenon could have great implication on human health. It should be noted that superinfection is possible in mosquitoes as well as in cells infected with heterologous viruses (i.e., different genus) and not with homologous ones [43]–[47]. Little is known about the molecular and cellular basis of co-infection which should be explored.

## Acknowledgments

We are grateful to the DRASS in the Reunion for providing the ALPROV mosquito strain and to the French National Reference Center for Arboviruses for the E1-226V CHIKV and the DENV-1 strains. We also wish to thank Sara Moutailler for critical reading of the manuscript. We are grateful to Michèle Bouloy and Félix Rey for their support.
